# Cytotoxic secondary metabolites isolated from *Penicillium* sp. YT2019-3321, an endophytic fungus derived from *Lonicera Japonica*

**DOI:** 10.3389/fmicb.2022.1099592

**Published:** 2022-12-13

**Authors:** Wenya Weng, Shicui Jiang, Chuchu Sun, Xiaofu Pan, Li Xian, Xuemian Lu, Chi Zhang

**Affiliations:** ^1^Department of Scientific Research, The Third Affiliated Hospital of Wenzhou Medical University, Zhejiang, China; ^2^Department of Endocrinology, Ruian People’s Hospital, Zhejiang, China; ^3^College of Life Sciences, Ludong University, Yantai, China

**Keywords:** polyketides, secondary metabolites, *Penicillium*, endophytic fungus, cytotoxic activity

## Abstract

**Introduction:**

Endophytic fungi associated with medicinal plants have proven to possess a high potential to produce structurally diverse metabolites, some of which are valuable for medicinal applications. In this study, *Penicillium* sp. YT2019-3321, an endophytic fungus derived from traditional Chinese medicine Lonicera japonica, was chemically studied.

**Methods:**

The chemical structures of the isolated compounds were established by a correlative interpretation of HRESIMS and NMR spectroscopic data. The optical resolution of (±)-**1** by chiral HPLC yielded individual enantiomers (+)**-1** and (–)**-1**, and their stereochemistry were solved by X-ray diffraction crystallography, respectively.

**Results and discussion:**

Eight structurally diversified secondary metabolites, including two previously unreported polyketides, named (±)-chrysoalide B (**1**) and penicidone E (**2**), were isolated and identified from Penicillium sp. YT2019-3321. Compound 2 possessed the γ-pyridone nucleus, which is rarely found in natural products. Cytotoxic assay revealed that the new compound 2 demonstrated a dose-dependent cytotoxicity against the human pancreatic tumor cells PATU8988T with the IC_50_ value of 11.4 μM. Further studies indicated that **2** significantly induced apoptosis of PATU8988T cell lines, characterized by the morphologies abnormity, the reduction of cell number, the upregulation of proportion of apoptotic cells, and the ratio of Bcl-2 to Bax. Our study demonstrates that fungal secondary metabolites may have important significance in the discovery of drug leads.

## 1 Introduction

Filamentous fungi from both marine and terrestrial sources are inherently regarded as a treasure house of structurally diversified secondary metabolites with potent pharmacological activity (Bills and Gloer, 2016; Zhang et al., 2016; Deshmukh et al., 2018). Fungi possess a well-developed secondary metabolism, which hold unique biosynthetic pathways to produce these fungal metabolites with a staggering variation in chemical structures and biological activities (Yu and Keller, 2005; Fox and Howlett, 2008; Ortega et al., 2021). Fungal metabolites have developed many important pharmaceuticals. The success of the β-lactam antibiotics including penicillins and cephalosporins effectively aroused the enthusiasm of the development of microbial medicines and contributed significantly in the establishment of the modern pharmaceutics (Zhang et al., 2020; Li et al., 2021). Subsequently, a large number of fungal-sourced pharmaceuticals with various mode of action, such as fusidic acid, griseofulvin, pneumocandin, lovastatin, cyclosporin A, and ergometrine, have been on the market (Bills and Gloer, 2016). It is estimated that an appreciable portion of natural-derived approved therapeutic agents were actually sourced from microorganisms, especially from fungi (Newman and Cragg, 2020). Moreover, many agricultural chemicals, including the existing fungicides, insecticides, and herbicides, are also fungal-derived (Sparks et al., 2017; Xu et al., 2021).

**FIGURE 1:**
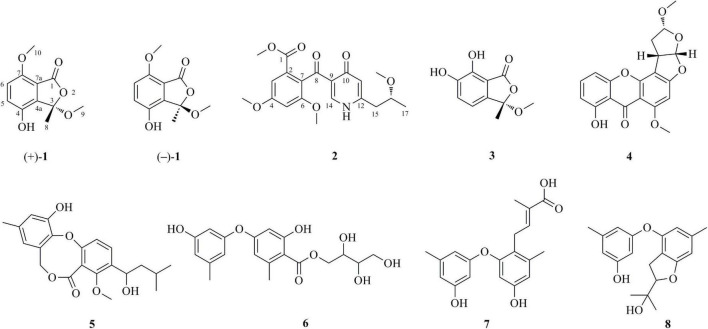
Structures of the isolated compounds **1-8**.

Endophytic fungi are recognized as microorganisms that spend the whole or part of their lifetime colonizing inter-and/or intra-cellularly plant tissues without causing any apparent disease symptoms (Aly et al., 2011). Endophytic fungi associated with medicinal plants have proven to possess a high potential to produce structurally diverse metabolites, some of which are valuable for medicinal and agricultural applications (Gouda et al., 2016). For example, chemical investigation of *Alternaria* sp. YUD20002, an endophytic fungus derived from the tubers of *Solanum tuberosum*, yielded five previously undescribed epoxy octa-hydronaphthalene polyketides altereporenes A-E (Xia et al., 2022). Acrocalysterols A and B, two new steroids were isolated from an endophytic fungus *Acrocalymma* sp. derived from the stems of *Sinomenium acutum* (Yang et al., 2022). Acrocalysterol B demonstrated strong cytotoxicity against HeLa, HCC-1806, and RKO cell lines with IC_50_ values of 18.37-19.64 μM (Yang et al., 2022). It should be pointed out that the genus belonging to *Penicillium* is considered as a rich resource of bioactive metabolites. In this study, chemical studies and chromatographic separation on *Penicillium* sp. YT2019-3321, an endophytic fungus derived from traditional Chinese medicine *Lonicera Japonica*, resulted in the isolation and identification of eight structurally diversified secondary metabolites, including two previously unreported polyketides, named (±)-chrysoalide B (**1**) and penicidone E (**2**) (Figure 1). The optical resolution of (±)-**1** by chiral HPLC yielded individual enantiomers (+)-**1** and (–)-**1**, and their stereochemistry were solved by X-ray diffraction crystallography. The new compound **2** possessed the γ-pyridone nucleus, which is rarely found in natural products. In addition to the structural elucidation, the cytotoxic activity of the isolated compounds is also described herein.

## 2 Materials and methods

### 2.1 General experimental procedures

Optical rotations were measured with a JASCO P-1020 digital polarimeter (Tokyo, Japan). UV spectra were obtained on a Lambda 35 UV/Vis spectrophotometer (Perkin Elmer, Waltham, United States). HRESIMS data were acquired with a scientific LTQ Orbitrap XL spectrometer (Thermo Scientific, Waltham, United States). 1D (500 and 125 MHz for ^1^H and ^13^C, respectively) and 2D (HSQC, COSY, and HMBC) NMR spectra were performed by an Agilent DD2 500 MHz spectrometer (Agilent Technologies, Santa Clara, United States). X-ray diffraction data were collected on an Agilent Xcalibur Gemini E diffractometer equipped with Eos charge-coupled device (CCD) detector with graphite monochromated Cu Kα radiation (λ = 1.54178 Å). Column chromatography was undertaken by using various packing materials including silica gel (100-200/200-300 mesh, Qingdao Marine Chemical Factory, Qingdao, China), octadecylsilyl (ODS) reversed-phase gel (30-50 μm, YMC CO., Ltd., Japan), and Sephadex LH-20 (GE Healthcare, United States).

### 2.2 Fungal material and fermentation

The fungal strain *Penicillium* sp. YT2019-3321 was previously isolated from the traditional Chinese medicine *Lonicera Japonica*. The taxonomic identification of this fungus was performed based on a molecular protocol by DNA amplification and sequencing of the internal transcribed spacer (ITS) of the rRNA locus. The ITS sequence showed 99% identical to that of *P. oxalicum* (GenBank accession no. KY400080.1). A voucher specimen of this fungal strain was stored at –80°C at the Third Affiliated Hospital of Wenzhou Medical University. This fungus was cultured on potato dextrose agar medium (PDA, Solarbio Life Sciences CO., Ltd., Beijing, China) at 28°C for 5 days. Then all of agar plugs were cut into small pieces (0.5 × 0.5 cm^2^). Each piece was inoculated in a 1 L Erlenmeyer flask containing 250 mL of potato dextrose broth (PDB) medium (Solarbio). A total of 100 flasks were statically fermented at room temperature for 30 days.

### 2.3 Extraction and isolation

The fermentation materials were adequately extracted with EtOAc (3 × 25 L), and the organic solvent was evaporated in vacuum to yield ca. 20 g of crude extracts. The crude extracts were subjected to a silica gel vacuum liquid chromatography column, which was eluted with an increasing gradient of EtOAc/petroleum ether (from 30:1 to 1:1) to afford six fractions (Fr. 1-Fr. 6). Fr. 4 (3.2 g), eluting with EtOAc/petroleum ether 5:1, was further fractionated over an ODS reversed-phase silica gel with a mixed solvent system of MeOH/H_2_O (from 10 to 100%, v/v). This afforded a total of eight subfractions (Fr. 4.1-Fr. 4.8). Fr. 4.6 was further purified over an open silica gel column chromatography by using the solvent system CH_2_Cl_2_ and MeOH with the ratio 20:1 to afford 16 mg of compound **2**. Fr. 5 (2.5 g), eluting with EtOAc/petroleum ether 2:1, was applied to ODS silica gel with gradient elution of MeOH/H_2_O (from 10 to 100%, v/v) to yield eight subfractions (Fr. 5.1-Fr. 5.8). Compound **1** (10.2 mg) was isolated from a two-step purification process, first from Fr. 5.3 over an open silica gel column chromatography using the solvent system CH_2_Cl_2_ and MeOH with the ratio 20:1, followed by preparative TLC (CH_2_Cl_2_/MeOH, 15:1, v/v). Compound **1** was further resolved into the pure enantiomers (+)-**1** (4.9 mg, *t*_*R*_ = 9.6 min) and (–)-**1** (4.7 mg, *t*_*R*_ = 10.9 min) by chiral HPLC using a (*R*,*R*). Whelk-O1 chiral column (10 mm; 4.6 × 250 mm; *n*-hexane-ethanol eluent 6:4, v/v; 1.0 mL/min). Compound **3** (10.2 mg, *t*_*R*_ 7.7 min) was isolated from Fr. 5.4 by semipreparative HPLC (YMC-pack ODS-A, 5 μm; 10 × 250 mm; 55% MeOH/H_2_O; flow rate 2 mL/min). Compound **7** (5.6 mg) was isolated from Fr. 5.5 by preparative TLC (CH_2_Cl_2_/MeOH/acetic acid, 15:1:0.4, v/v). Compound **6** (11.3 mg) was isolated from Fr. 5.6 by preparative TLC (CH_2_Cl_2_/MeOH/acetic acid, 20:1:0.4, v/v). Fr. 6 (4.0 g), eluting with EtOAc/petroleum ether 1:1, was fractionated by Sephadex LH-20 column chromatography in MeOH to give subfractions Fr. 6.1-Fr. 6.3. Fr. 6.1 was subjected to semipreparative HPLC (65% MeOH/H_2_O) to give compounds **4** (20.2 mg, *t*_*R*_ 6.9 min) and **5** (6.2 mg, *t*_*R*_ 8.8 min), respectively. Finally, compound **8** (4.9 mg) was obtained by preparative TLC (CH_2_Cl_2_/MeOH, 20:1, v/v) from Fr. 6.3.

(±)-Chrysoalide B (**1**): white amorphous powder; [α]^20^_*D*_ + 9.6 (*c* 0.10, MeOH) for (+)-**1** and [α]^20^_*D*_ –10.2 (*c* 0.10, MeOH) for (–)-**1**; UV (MeOH) λ_*max*_ (log ε) 213 (2.16), 239 (1.60), 331 (1.49) nm; ^1^H and ^13^C NMR data (measured in DMSO-*d*_6_) (see Table 1); HRESIMS *m/z* 223.0644 [M - H]^–^ (calcd for C_11_H_11_O_5_, 223.0606).

**TABLE 1:** NMR data for compounds (±)-**1** and **2** in DMSO-*d*_6_ (^1^H at 500 MHz and ^13^C at 125 MHz).

No.	Compound (±)-1		No.	Compound 2	
	δ_H_ (mult, *J* in Hz)	δ_C_, type		δ_H_ (mult, *J* in Hz)	δ_C_, type
1		165.1, C	1		166.3, C
3		106.8, C	2		129.6, C
4		146.6, C	3	6.93 (s)	105.3, CH
4a		132.2, C	4		160.0, C
5	7.13 (d, 8.8)	123.5, CH	5	6.80 (s)	103.2, CH
6	7.05 (d, 8.8)	115.2, CH	6		157.6, C
7		150.2, C	7		127.9, C
7a		115.2, C	8		192.4, C
8	1.75 (s)	23.6, CH_3_	9		124.5, C
9	2.94 (s)	50.7, CH_3_	10		175.8, C
10	3.80 (s)	55.9, CH_3_	11	6.00 (s)	121.3, CH
			12		148.5, C
			14	8.12 (s)	142.2, CH
			15	2.60 (m)	39.2, CH_2_
			16	3.62 (m, overlap)	75.3, CH
			17	1.11 (d, 6.1)	19.1, CH_3_
			1-OMe	3.64 (s)	52.5, CH_3_
			4-OMe	3.84 (s)	56.0, CH_3_
			6-OMe	3.66 (s)	56.5, CH_3_
			16-OMe	3.23 (s)	56.0, CH_3_

Penicidone E (**2**): colorless oil; [α]^20^_*D*_ + 13.5 (*c* 0.10, MeOH); UV (MeOH) λ_*max*_ (log ε) 220 (3.88), 254 (3.26), 309 (2.98); ^1^H and ^13^C NMR data (measured in DMSO-*d*_6_) (see Table 1); HRESIMS *m/z* 390.1547 [M + H]^+^ (C_20_H_24_NO_7_) and 412.1369 [M + Na]^+^ (C_20_H_23_NO_7_Na).

### 2.4 X-ray crystallographic analysis of (+)-1 and (–)-1

Suitable crystals of (+)-**1** and (–)-**1** were obtained by slowly evaporating the solvent mixture of MeOH and H_2_O. Single-crystal X-ray diffraction data were obtained on an Agilent Xcalibur Gemini E diffractometer equipped with Eos CCD detector with graphite monochromated Cu Kα radiation (λ = 1.54178 Å). Structures were solved by direct methods using the SHELXTL software package (Sheldrick, 1997a). All non-hydrogen atoms were refined anisotropically. H atoms were located by geometrical calculations, and their positions and thermal parameters were fixed during structure refinement. Structure was refined by full-matrix least-squares techniques (Sheldrick, 1997b).

Crystal data for (+)-**1**: C_22_H_26_O_11_ (2 C_11_H_12_O_5_ + H_2_O), F.W. = 466.43, monoclinic space group P2_1_, unit cell dimensions *a* = 7.3590 (9) Å, *b* = 14.4044 (16) Å, *c* = 10.4189 (12) Å, α = β = γ = 90°, *V* = 1103.9 (2) Å^3^, *Z* = 2, *d*_*calcd*_ = 1.403 mg/m^3^. Crystal size: 0.08 × 0.05 × 0.04 mm^3^, μ = 0.967 mm^–1^, *F* (000) = 492.0. Reflections collected/unique: 19,174/4,377 [*R* (int) = 0.0438]. Final indices resulted in *R*_1_ = 0.0424 and *wR*_2_ = 0.1067 [*I* > 2σ(*I*)] Flack parameter = 0.13 (6).

Crystal data for (–)-**1**: C_22_H_26_O_11_ (2 C_11_H_12_O_5_ + H_2_O), F.W. = 466.43, monoclinic space group P2_1_, unit cell dimensions *a* = 7.3638 (2) Å, *b* = 14.3632 (4) Å, *c* = 10.4176 (3) Å, α = β = γ = 90°, *V* = 1101.11 (5) Å^3^, *Z* = 2, *d*_*calcd*_ = 1.407 mg/m^3^. Crystal size: 0.12 × 0.07 × 0.04 mm^3^, μ = 0.970 mm^–1^, *F* (000) = 492.0. Reflections collected/unique: 25,073/4,450 [*R* (int) = 0.0590]. Final indices resulted in *R*_1_ = 0.0358 and *wR*_2_ = 0.0827 [*I* > 2σ(*I*)] Flack parameter = 0.05 (10).

### 2.5 Cytotoxic bioassay

#### 2.5.1 Cell culture

The human pancreatic cancer cell line PATU8988T was acquired from Shanghai Fuheng Biotechnology Co., Ltd., RPMI 1640 medium containing 10% fetal bovine serum (Gibco, Gaithersburg, MD, USA) was used. The cells were cultured in 5% CO_2_ at 37°C. Cells were treated with the positive control doxorubicin (dox) at the dose of 10 μM and the test compounds at the dose of 20 μM, respectively, for 48 h when they reached ∼80% confluence.

#### 2.5.2 Cell viability assay

CCK-8 (Solarbio) was applied to detect the cell viability according to the manufacturer’s instruction as previously described (Yuan et al., 2020). In brief, cells were treated with test compounds at the gradient concentration of 1, 5, 10, 20, 30, 40, and 50 μM for 24 and 48 h, respectively. Doxorubicin (dox) at the concentration of 10 μM was applied as the positive control. Then the media of the cells was changed with 10% CCK-8 solution followed by indcubating 5% CO_2_ at 37°C. Cell viability was detected at absorbance of 450 nm.

#### 2.5.3 Flow cytometry

Cell apoptosis was examined by flow cytometry using Annexin V-FITC Apoptosis Detection Kit (Beyotime Biotechnology, China) according to the manufacturer’s instruction. Cells were incubated with or without test compounds at 20 μM for 48 h, followed by being treated with 200 mL binding buffer and stained with Annexin V-FITC and PI for 40 min in the dark. After that, the cells were assessed by flow cytometry (Agilent, United States).

#### 2.5.4 Western blot analysis

RIPA buffer (Beyotime Biotechnology, China) containing protease inhibitors (Beyotime Biotechnology, China) was applied to extract protein lysates of the cells. The concentration of protein was examined by the Bradford assay. The samples were diluted in loading buffer and denatured at 95°C for 5 min. Then they were separated in SDS PAGE gel followed by being transferred into nitrocellulose membranes for next steps. After being treated with blocking solution for 1 h at room temperature, the membranes were incubated with the following primary antibodies: Bax and Bcl-2 purchased from ABclone. After that, membranes were washed with Tris-buffered saline (pH 7.2) containing 0.05% Tween 20 for 15 min for three times followed by being treated with secondary antibodies for 1 h at room temperature. Bands were visualized with ECL substrate (Bio-Rad Laboratories).

### 2.6 Computational details

The conformer rotamer ensemble sampling tool (crest) (Pracht et al., 2020) was used to afford candidate conformers for *S*-**2** and DFT calculations were performed with the Gaussian 16 program (Frisch et al., 2016). The conformers within an energy window of 10 kcal/mol were optimized at B3LYP/6-31G (d) level of theory with Grimme’s D3 dispersion correction (“EmpiricalDispersion = GD3” key words in input files). Frequency analysis of all optimized conformations was undertaken at the same level of theory to ensure they were true local minima on the potential energy surface. Then, energies of all optimized conformations were evaluated by M062X/6-311 + G (2d,p) with D3 dispersion correction. Gibbs free energies of each conformers were calculated by adding “Thermal correction to Gibbs Free Energy” obtained by frequency analysis to electronic energies obtained at M062X/6-311 + G (2d,p). Room-temperature (298.15 K) equilibrium populations were calculated according to Boltzmann distribution law. Those conformers accounting for over 2% population were subjected to subsequent calculations. Calculation of optical rotations of different conformers were carried out using the TDDFT method at CAM-B3LYP/6-311 + g (2d,p) level in methanol (λ = 589 nm). Detailed computational data have shown in Supplementary material.

## 3 Results and discussion

### 3.1 Structural elucidation

**FIGURE 2:**
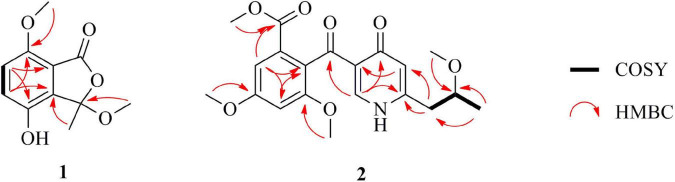
Key COSY and HMBC correlations for **1** and **2**.

(±)-Chrysoalide B (**1**) was isolated as white amorphous powder. Its molecular formula, C_11_H_12_O_5_, was established by HRESIMS at *m/z* 223.0644 [M-H]^–^ (calcd for C_11_H_11_O_5_, 223.0606). Observation of the ^1^H NMR data of **1** (Table 1) revealed the presence of three methyl groups including two methoxy groups at δ_*H*_ 2.94 (s, H_3_-9) and 3.80 (s, H_3_-10) as well as two coupled aromatic methines at δ_*H*_ 7.13 (d, *J* = 8.8 Hz, H-5) and 7.05 (d, *J* = 8.8 Hz, H-6). The ^13^C spectroscopic data of **1** (Table 1) displayed 11 carbon resonances, including an ester carbonyl at δ_*C*_ 165.1 (C-1), five quaternary carbons including one oxygenated sp^3^ at δ_*C*_ 106.8 (C-3), two aromatic methines at δ_*C*_ 123.5 (C-5) and 115.2 (C-6), and three methyl groups including two methoxy groups at δ_*C*_ 50.7 (C-9) and 55.9 (C-10). Considering the functional groups observed for compound **1** as well as the characteristic UV absorption peaks at 213, 239, and 331 nm (Saetang et al., 2021), the presence of an isobenzofuran core framework was deduced. HMBC correlations (Figure 2) from H-5 to C-4a and C-7 as well as from H-6 to C-4 and C-7a deduced the presence of a 1,2,3,4-tetrasubstituted benzene group. Finally, HMBC correlations from H_3_-9 to C-3 and from H_3_-10 to C-7 led to the location of two methoxy groups at C-3 and C-7, respectively. The planar structure of **1** was thus determined as shown in Figure 1.

**FIGURE 3:**
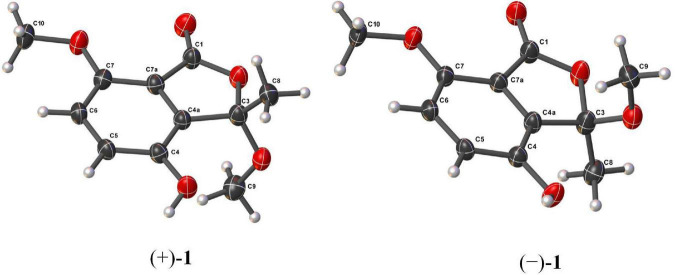
Molecular structures of (+)-**1** and (–)-**1** from single-crystal X-ray diffractometry.

Compound **1** possessed the same planar structure as chrysoalide B, a new phthalide produced by the marine-derived fungus *Penicillium chrysogenum* LD-201810 (Ge et al., 2021). Surprisingly, the specific rotation value of **1** was found to be zero. Considering its baseline ECD curve, compound **1** was existed as racemic enantiomers. Compound **1** was then subjected to chiral HPLC separation, which successfully afforded two individual enantiomers (+)-**1** and (–)-**1** with a ratio of 1:1. Both (+)-**1** and (–)-**1** were cultured into suitable single crystals in MeOH/H_2_O mixed solution. Single-crystal X-ray diffraction of (+)-**1** and (–)-**1** (Figure 3) not only confirmed the proposed structure but also the absolute configurations of (+)-**1** and (–)-**1**. It should be pointed out that Ge et al. (2021) reported the new compound chrysoalide B with the optical rotation of + 15.8° and established the absolute configuration of C-3 to be *R* based on ECD calculations. However, in our study, the absolute configuration of (+)-chrysoalide B (**1**) was revised as 3*S* by single-crystal X-ray diffraction.

Penicidone E (**2**), isolated as colorless oil, was found to possess the molecular formula of C_20_H_23_NO_7_ on the basis of HRESIMS (*m/z* 390.1547 [M + H] ^+^ for C_20_H_24_NO_7_ and 412.1369 [M + Na] ^+^ for C_20_H_23_NO_7_Na). Overall inspection of the ^1^H and ^13^C NMR spectra of **2** (Table 1) indicated that it contained two carbonyls at δ_*C*_ 192.4 (C-8) and 175.8 (C-10), one ester carbonyl at δ_*C*_ 166.3 (C-1), ten sp^2^-hybridized carbons which resonated between δ_*C*_ 103.2 and 160.0, one methylene at δ_*C*_ 39.2 (C-15), one oxygenated sp^3^ methine at δ_*C*_ 75.3 (C-16), and five methyls including four methoxy groups at δ_*C*_ 52.5 (1-OMe), 56.0 (4-OMe), 56.5 (6-OMe), and 56.0 (16-OMe). The ^1^H and ^13^C NMR spectra of **2** were partially similar to that of penicidone C, a cytotoxic alkaloidal metabolite isolated from an endophytic *Penicillium* sp. (Ge et al., 2008). However, the resonances at δ_*C*_ 125.3 and 134.2 ascribable to the two sp^2^ methine groups in penicidone C were replaced by a methylene (C-15) and an oxygenated sp^3^ methine (C-16). Moreover, an extra methoxyl signal at δ_*H*_ 3.23 and δ_*C*_ 56.0 (16-OMe) appeared in the ^1^H and ^13^C NMR spectra of **2**. The observation could be explained by assuming that **2** was an oxidative derivative of penicidone C at the location of C-15 and C-16. This assumption was reinforced by the HMBC correlations from H_3_-17 to C-15 and C-16, from H_2_-15 to C-11 and C-12, and from 16-OMe to C-16 (Figure 2). Compound **2** was named as penicidone E. The γ-pyridone nucleus found in **2** is rare in natural products, with only four analogs, penicidones A-D, possessing similar structures (Liu et al., 2015). Calculation of optical rotations of different conformers 16*R* and 16*S* were carried out using the TDDFT method at CAM-B3LYP/6-311 + g (2d,p) level in methanol (λ = 589 nm). The calculated optical rotation was –24.4, which was of the opposite sign to the experimental value ([α]^20^_*D*_ + 13.5). Therefore, the absolute configuration of **2** was established as 16*R*.

In addition to the new compounds **1** and **2**, the structures of the remaining six known compounds were established based on their spectroscopic data, as well as by comparison with the literatures. These compounds were identified as 6,7-dihydroxy-3-methoxy-3-methylphthalide (**3**) (Wang et al., 2013), oxisterigmatocystin C (**4**) (Cai et al., 2011), penicillide (**5**) (Jeon and Shim, 2020), a diphenyl ether **6** (Wu et al., 2018), diorcinol L (**7**) (Li et al., 2018), and 3-[2-(1-hydroxy-1-methyl-ethyl)-6-methyl-2,3-dihydrobenzofuran-4-yloxy]-5-methylphenol (**8**) (Zhuravleva et al., 2013).

### 3.2 Cytotoxic activity

**FIGURE 4:**
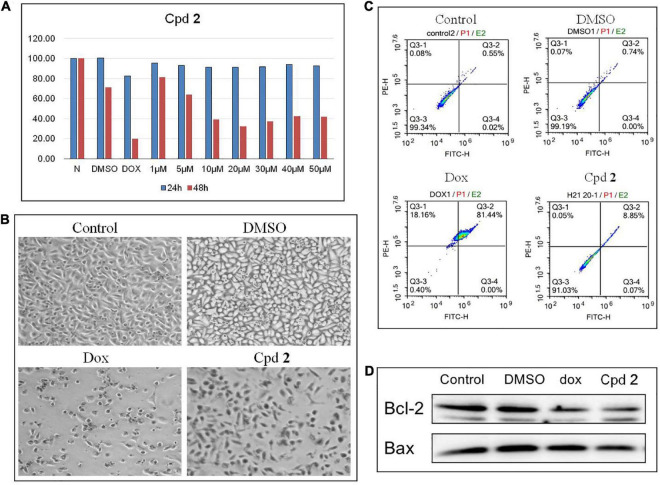
Compound **2** (Cpd **2**) induced human pancreatic cancer cells apoptosis. **(A)** Cytotoxic effect of Cpd **2** on PATU8988T measured by CCK-8. Cells were treated with Cpd **2** at the gradient concentrations from 1 to 50 μM for 24 and 48 h, respectively. **(B)** Morphological results of PATU8988T treated with Cpd **2**. **(C)** Annexin V/PI double staining with flow cytometry analysis was used for the detection of cell apoptosis. **(D)** Western blotting of Bcl-2 and Bax.

The isolated compounds **1**-**8** were detected for their cytotoxicity against human pancreatic cancer cell line PATU8988T by using the CCK-8 method (Yuan et al., 2020). The results of CCK-8 showed that only the new compound **2** showed a promising activity (Supplementary Table 6). **2** demonstrated a dose-dependent cytotoxicity against the cells treated for 48 h, with the IC_50_ value of 11.4 μM (Figure 4A). Cell apoptosis might inhibit tumor cell proliferation. Therefore, compounds which could aggravate tumor cell apoptosis might possess application prospect for the anti-tumor therapy. To further study whether the inhibition of PATU8988T cells proliferation was caused by cell apoptosis, we examined apoptosis-related indicators. After treated with compound **2** at the concentration of 20 μM for 48 h, PATU8988T cell number reduction and cell morphology abnormity occurred in both doxorubicin (dox) and **2**-treated groups while the cells in the control group and DMSO group performed normally (Figure 4B), suggesting **2** as well as dox induced PATU8988T cell death. Additionally, Annexin V-FITC/PI assay was used to examine apoptosis percentage by flow cytometry. As shown in Figure 4C, dox significantly up-regulated the proportion of apoptotic cells with a percentage of 81.44% while **2** increased the proportion of apoptotic cells with a percentage of 8.85% in comparison with 0.55% in the control group and 0.74% in DMSO group. In addition, Bcl-2 family members including Bcl-2 and Bax can regulate apoptosis. The result of western blotting demonstrated that both dox and **2** markedly decreased the ratio of Bcl-2/Bax, indicating that **2** might induce pancreatic tumor cell apoptosis (Figure 4D). The above results proved that **2** might kill pancreas tumor cells by inducing the cell apoptosis.

## 4 Conclusion

Eight structurally diversified secondary metabolites, including two previously unreported polyketides, named (±)-chrysoalide B (**1**) and penicidone E (**2**), were isolated from *Penicillium* sp. YT2019-3321, an endophytic fungus derived from traditional Chinese medicine *Lonicera Japonica*. The optical resolution of (±)-**1** by chiral HPLC yielded individual enantiomers (+)-**1** and (–)-**1**, and their stereochemistry were solved by X-ray diffraction crystallography, respectively. The γ-pyridone nucleus found in **2** is rare in natural products, with only four analogs having similar structures. Tumor cell proliferation might be inhibited by cell apoptosis. Our study demonstrated that the new compound **2** significantly induced apoptosis in human pancreatic tumor cells (PATU8988T), characterized by the morphologies abnormity, the reduction of cell number, the upregulation of proportion of apoptotic cells, and decrease in the ratio of Bcl-2 to Bax.

## Data availability statement

The datasets presented in this study can be found in online repositories. The names of the repository/repositories and accession number(s) can be found in the article/Supplementary material.

## Author contributions

CZ and XL: conception or design. SJ, WW, CS, XP, and LX: acquisition, analysis, and interpretation of data. WW: drafting the work and revising. CZ, XL, and WW: final approval of the manuscript. All authors contributed to the article and approved the submitted version.
