# Supplementary material for: Cytotoxic secondary metabolites isolated from Penicillium sp. YT2019-3321, an endophytic fungus derived from Lonicera Japonica
Source: Front Microbiol. 2022 Dec 13;13:1099592. doi: 10.3389/fmicb.2022.1099592 (PMC9792606; doi:10.3389/fmicb.2022.1099592)
Supplement: Supplementary file 1 [file Data_Sheet_1.docx]

**Supplementary Material**

Cytotoxic secondary metabolites isolated from *Penicillium* sp. YT2019-3321, an endophytic fungus derived from *Lonicera Japonica*

Wenya Weng^1, #^, Shicui Jiang^1, #^, Chuchu Sun^1^, Xiaofu Pan^1^, Li Xian^3^, Xuemian Lu^1, 2^*, Chi Zhang^1,^*

^1^The Third Affiliated Hospital of Wenzhou Medical University, Zhejiang 325200, China

^2^Department of Endocrinology, Ruian people’s Hospital, Zhejiang 325200, China

^3^College of Life Sciences, Ludong University, Yantai 264025, China

***Correspondence:**

Xuemian Lu: luxuemian@wmu.edu.cn

Chi Zhang: zhangchi515@126.com

^#^These authors contributed equally to this work.

Keywords: Polyketides, Secondary metabolites, *Penicillium*, Endophytic fungus, Cytotoxic activity

**Contents**

**Figure S1.** HRESIMS spectrum of compound **1**

**Figure S2.** ^1^H NMR (500 MHz, DMSO-*d*_6_) spectrum of compound **1**

**Figure S3.** ^13^C NMR (125 MHz, DMSO-*d*_6_) spectrum of compound **1**

**Figure S4.** HSQC spectrum of compound **1**

**Figure S5.** COSY spectrum of compound **1**

**Figure S6.** HMBC spectrum of compound **1**

**Figure S7.** HRESIMS spectrum of compound **2**

**Figure S8.** ^1^H NMR (500 MHz, DMSO-*d*_6_) spectrum of compound **2**

**Figure S9.** ^13^C NMR (125 MHz, DMSO-*d*_6_) spectrum of compound **2**

**Figure S10.** HSQC spectrum of compound **2**

**Figure S11.** COSY spectrum of compound **2**

**Figure S12.** HMBC spectrum of compound **2**

**Original computational data for compound 2**

**Cytotoxic results of compounds 1−8 against PATU8988T cell line**

**
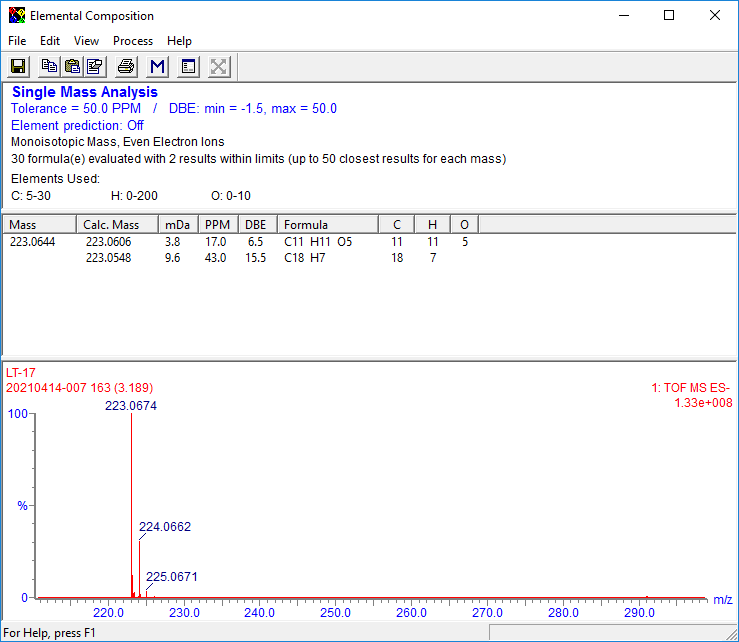
**

**Figure S1.** HRESIMS spectrum of compound **1**

**Figure S2.** ^1^H NMR (500 MHz, DMSO-*d*_6_) spectrum of compound **1**

**Figure S3.** ^13^C NMR (125 MHz, DMSO-*d*_6_) spectrum of compound **1**

**Figure S4.** HSQC spectrum of compound **1**

**Figure S5.** COSY spectrum of compound **1**

**Figure S6.** HMBC spectrum of compound **1**

**
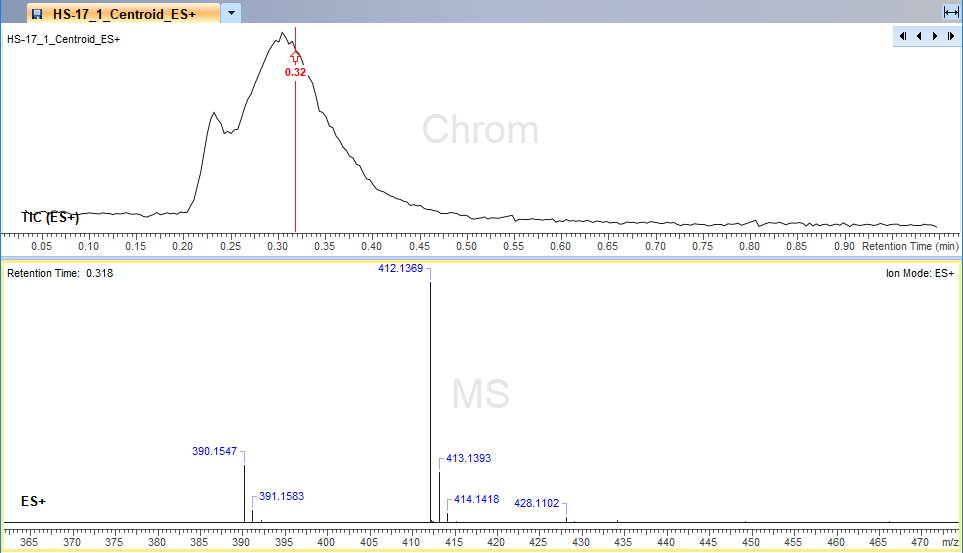
Figure S7.** HRESIMS spectrum of compound **2**

**Figure S8.** ^1^H NMR (500 MHz, DMSO-*d*_6_) spectrum of compound **2**

**Figure S9.** ^13^C NMR (125 MHz, DMSO-*d*_6_) spectrum of compound **2**

**Figure S10.** HSQC spectrum of compound **2**

**Figure S11.** COSY spectrum of compound **2**

**Figure S12.** HMBC spectrum of compound **2**

**Original computational data for compound 2**

Table S1. Conformational analysis of the B3LYP/6-31G(d) optimized conformers of *S*-**2** in the gas phase (T=298.15 K)

| **Conformer** | **E*^a^* (Hartree)** | **C*^b^* (Hartree)** | **G*^c^* (kcal/mol)** | **ΔG*^d^* (kcal/mol)** | **Population*^e^*** |
| --- | --- | --- | --- | --- | --- |
| *S*-**2**-**1** | -1357.155377 | 0.342161 | -851400.293153 | 0.0 | 47.42% |
| *S*-**2**-**2** | -1357.155863 | 0.343012 | -851400.06409 | 0.229063 | 32.21% |
| *S*-**2**-**3** | -1357.155681 | 0.343519 | -851399.631755 | 0.661398 | 15.52% |
| *S*-**2**-**4** | -1357.153491 | 0.342424 | -851398.944386 | 1.348767 | 4.86% |

*^a^*Electronic energy obtained at M062X/6-311+G(2d,p) level of theory; *^b^*Thermal correction to Gibbs free energy obtained at B3LYP/6-31G(d) level of theory; *^c^*Gibbs free energy (E + C); *^d^*The relative Gibbs free energy; *^e^*The Boltzmann distribution of each conformer.

Table S2. Atomic coordinates (Å) of *S*-**2**-**1** obtained at the B3LYP/6-31G(d) level of theory in the gas phase.

| C | 2.082578 | 4.528151 | -0.036260 | C | 6.313289 | 0.311530 | -1.453528 |
| --- | --- | --- | --- | --- | --- | --- | --- |
| O | 2.684525 | 3.229202 | 0.021049 | C | 3.771696 | 0.743901 | -0.330487 |
| C | 1.817605 | 2.208364 | 0.228962 | H | 2.904212 | 5.224237 | -0.208303 |
| O | 0.622647 | 2.383590 | 0.369287 | H | 1.355795 | 4.580360 | -0.851669 |
| C | 2.492394 | 0.883585 | 0.236238 | H | 1.574444 | 4.756681 | 0.904278 |
| C | 1.791906 | -0.222154 | 0.716284 | H | -1.952512 | 0.267653 | 2.253365 |
| C | 0.454209 | -0.115586 | 1.403124 | H | -4.020599 | -0.141584 | 1.148670 |
| O | 0.399634 | 0.287588 | 2.557186 | H | -4.600107 | -1.600214 | -2.087641 |
| C | -0.772665 | -0.516763 | 0.663921 | H | -5.157406 | -2.037991 | -0.470203 |
| C | -1.954379 | -0.210456 | 1.280180 | H | -6.515462 | -0.252374 | -1.485697 |
| N | -3.160192 | -0.470335 | 0.720512 | H | -4.700988 | 0.955699 | -2.738260 |
| C | -3.264209 | -1.034936 | -0.536133 | H | -4.009586 | 1.507347 | -1.202632 |
| C | -4.662668 | -1.250561 | -1.053484 | H | -5.620488 | 2.059960 | -1.709414 |
| C | -5.558394 | -0.002616 | -1.000675 | H | -7.027847 | 1.154019 | 1.730085 |
| C | -4.936971 | 1.205960 | -1.698614 | H | -6.493682 | 2.220717 | 0.406336 |
| O | -5.819598 | 0.235470 | 0.391912 | H | -7.741675 | 0.977674 | 0.103481 |
| C | -6.819498 | 1.203366 | 0.658841 | H | -2.192768 | -1.814028 | -2.192101 |
| C | -2.131218 | -1.365729 | -1.206289 | H | 1.771081 | -3.740929 | -0.548952 |
| C | -0.786305 | -1.156854 | -0.662191 | H | 2.799618 | -4.239692 | 0.839266 |
| O | 0.219837 | -1.505794 | -1.292383 | H | 1.030182 | -4.426313 | 0.928479 |
| C | 2.385091 | -1.490559 | 0.617062 | H | 4.110031 | -2.619530 | -0.047324 |
| O | 1.644186 | -2.517500 | 1.119675 | H | 7.248186 | -0.108149 | -1.829016 |
| C | 1.837847 | -3.801597 | 0.542397 | H | 5.786795 | 0.819718 | -2.272030 |
| C | 3.641585 | -1.647947 | 0.048413 | H | 6.532009 | 1.039899 | -0.661645 |
| C | 4.337239 | -0.524095 | -0.421458 | H | 4.276545 | 1.623186 | -0.702511 |
| O | 5.565116 | -0.785560 | -0.957721 | - | - | - | - |

Table S3. Atomic coordinates (Å) of *S*-**2**-**2** obtained at the B3LYP/6-31G(d) level of theory in the gas phase.

| C | 1.590507 | 4.069755 | 0.588267 | C | 6.573469 | -1.274175 | 0.548838 |
| --- | --- | --- | --- | --- | --- | --- | --- |
| O | 1.725725 | 2.650249 | 0.458584 | C | 4.179239 | 0.116614 | 0.057691 |
| C | 2.924110 | 2.222563 | 0.008152 | H | 1.727318 | 4.554769 | -0.382016 |
| O | 3.877357 | 2.958277 | -0.153097 | H | 0.577851 | 4.232393 | 0.958299 |
| C | 2.952743 | 0.745137 | -0.195793 | H | 2.327871 | 4.463610 | 1.292572 |
| C | 1.816995 | 0.010614 | -0.545794 | H | -1.815936 | 1.501208 | -1.628366 |
| C | 0.529697 | 0.670721 | -0.942430 | H | -3.922883 | 1.103856 | -0.593472 |
| O | 0.519551 | 1.453583 | -1.887214 | H | -4.647308 | -0.725733 | 2.419721 |
| C | -0.722468 | 0.368628 | -0.194258 | H | -4.950757 | 0.945327 | 1.937259 |
| C | -1.862437 | 0.916684 | -0.716210 | H | -6.659152 | -0.604317 | 1.083327 |
| N | -3.078597 | 0.770493 | -0.137807 | H | -4.429234 | -1.743856 | -0.699336 |
| C | -3.242039 | 0.035177 | 1.020722 | H | -6.148561 | -2.186450 | -0.765239 |
| C | -4.643843 | -0.041391 | 1.566912 | H | -5.183708 | -2.601150 | 0.656319 |
| C | -5.699970 | -0.504358 | 0.550957 | H | -7.005080 | 1.399715 | -1.819853 |
| C | -5.346286 | -1.835622 | -0.109615 | H | -6.797646 | -0.359584 | -2.012378 |
| O | -5.831397 | 0.565756 | -0.397828 | H | -7.865079 | 0.278759 | -0.728854 |
| C | -6.931338 | 0.450697 | -1.283930 | H | -2.261917 | -1.119792 | 2.504562 |
| C | -2.155337 | -0.542345 | 1.592558 | H | 0.787019 | -3.435226 | 0.491281 |
| C | -0.801385 | -0.433280 | 1.040613 | H | -0.395819 | -3.645781 | -0.833617 |
| O | 0.157788 | -0.984684 | 1.592105 | H | 1.311837 | -4.074846 | -1.105708 |
| C | 1.931794 | -1.387846 | -0.640071 | H | 3.236696 | -3.103800 | -0.433563 |
| O | 0.807701 | -2.044693 | -1.046346 | H | 6.445182 | -0.709571 | 1.481188 |
| C | 0.635287 | -3.380048 | -0.591618 | H | 7.352769 | -2.026100 | 0.683551 |
| C | 3.134289 | -2.027731 | -0.369992 | H | 6.865490 | -0.583100 | -0.252025 |
| C | 4.262898 | -1.271264 | -0.023979 | H | 5.024286 | 0.735657 | 0.325976 |
| O | 5.395948 | -1.991066 | 0.211159 | - | - | - | - |

Table S4. Atomic coordinates (Å) of *S*-**2**-**3** obtained at the B3LYP/6-31G(d) level of theory in the gas phase.

| C | -0.015593 | 3.194266 | 1.407214 | C | 6.496825 | 0.822212 | -1.163819 |
| --- | --- | --- | --- | --- | --- | --- | --- |
| O | 0.775248 | 2.006905 | 1.309678 | C | 3.877632 | 0.862382 | -0.144327 |
| C | 1.914106 | 2.126675 | 0.593185 | H | 0.541404 | 3.988306 | 1.910840 |
| O | 2.354127 | 3.195855 | 0.223525 | H | -0.303388 | 3.530914 | 0.407632 |
| C | 2.568038 | 0.811273 | 0.348794 | H | -0.894309 | 2.911059 | 1.987781 |
| C | 1.910773 | -0.402025 | 0.557766 | H | -1.815764 | -1.462717 | 1.719703 |
| C | 0.531732 | -0.555766 | 1.155070 | H | -3.850049 | -1.271098 | 0.497435 |
| O | 0.423809 | -0.978698 | 2.297492 | H | -4.438597 | 0.346256 | -2.663469 |
| C | -0.678016 | -0.348180 | 0.305081 | H | -4.660645 | -1.317524 | -2.116757 |
| C | -1.822654 | -0.932973 | 0.773686 | H | -6.546179 | 0.134895 | -1.494912 |
| N | -2.996283 | -0.893789 | 0.097060 | H | -4.568546 | 1.529973 | 0.400650 |
| C | -3.109690 | -0.235619 | -1.111872 | H | -6.315452 | 1.838448 | 0.301315 |
| C | -4.460300 | -0.293165 | -1.776781 | H | -5.265609 | 2.258006 | -1.057066 |
| C | -5.632224 | 0.134844 | -0.879926 | H | -7.835259 | -0.725997 | 0.273505 |
| C | -5.436101 | 1.520154 | -0.266163 | H | -6.997133 | -1.742341 | 1.478091 |
| O | -5.768063 | -0.896120 | 0.110855 | H | -6.911674 | 0.033495 | 1.601782 |
| C | -6.939112 | -0.814220 | 0.904909 | H | -2.088980 | 0.916733 | -2.571211 |
| C | -2.019916 | 0.389462 | -1.625904 | H | 2.827968 | -4.043208 | -0.920938 |
| C | -0.717825 | 0.415044 | -0.954473 | H | 3.426520 | -4.114137 | 0.763663 |
| O | 0.228824 | 1.058589 | -1.423024 | H | 1.807294 | -4.752882 | 0.362904 |
| C | 2.598571 | -1.591421 | 0.239614 | H | 4.443645 | -2.455767 | -0.508648 |
| O | 1.892606 | -2.745422 | 0.418544 | H | 6.617796 | 1.402226 | -0.240008 |
| C | 2.537219 | -3.974122 | 0.135675 | H | 7.478888 | 0.544214 | -1.550164 |
| C | 3.899825 | -1.555837 | -0.252552 | H | 5.970713 | 1.435934 | -1.905753 |
| C | 4.541216 | -0.323462 | -0.439682 | H | 4.321928 | 1.836358 | -0.295631 |
| O | 5.813745 | -0.398830 | -0.923711 | - | - | - | - |

Table S5. Atomic coordinates (Å) of *S*-**2**-**4** obtained at the B3LYP/6-31G(d) level of theory in the gas phase.

| C | 1.598122 | 4.187635 | -0.779957 | C | 5.791143 | -2.979933 | -0.253986 |
| --- | --- | --- | --- | --- | --- | --- | --- |
| O | 1.786183 | 2.774928 | -0.640151 | C | 4.264791 | 0.322965 | -0.132523 |
| C | 2.965042 | 2.403210 | -0.096415 | H | 1.631243 | 4.672100 | 0.199671 |
| O | 3.871123 | 3.177607 | 0.131272 | H | 2.373242 | 4.618172 | -1.419171 |
| C | 3.036633 | 0.927168 | 0.119175 | H | 0.613617 | 4.306800 | -1.233268 |
| C | 1.913913 | 0.166319 | 0.485497 | H | -1.723689 | 1.582468 | 1.620148 |
| C | 0.621015 | 0.813637 | 0.872807 | H | -3.858012 | 1.060757 | 0.690286 |
| O | 0.615838 | 1.672041 | 1.750916 | H | -5.001359 | 0.748413 | -1.742001 |
| C | -0.647785 | 0.422117 | 0.194756 | H | -4.630160 | -0.910658 | -2.211847 |
| C | -1.785595 | 0.952047 | 0.740106 | H | -5.167692 | -1.590976 | 0.204760 |
| N | -3.018240 | 0.732101 | 0.223788 | H | -6.856130 | -1.892121 | -1.610090 |
| C | -3.202457 | -0.057524 | -0.893285 | H | -7.379753 | -0.202722 | -1.417879 |
| C | -4.621982 | -0.213471 | -1.369809 | H | -7.667371 | -1.383611 | -0.125018 |
| C | -5.600310 | -0.710704 | -0.296269 | H | -7.236330 | -0.453373 | 1.871725 |
| C | -6.957947 | -1.068164 | -0.895964 | H | -5.565818 | -0.836972 | 2.376566 |
| O | -5.723592 | 0.349832 | 0.667368 | H | -6.211235 | 0.818206 | 2.577021 |
| C | -6.212042 | -0.064768 | 1.933885 | H | -2.240395 | -1.234835 | -2.372525 |
| C | -2.118718 | -0.617274 | -1.489074 | H | 0.917819 | -3.274833 | -0.501583 |
| C | -0.748047 | -0.433445 | -1.002829 | H | 1.494909 | -3.932414 | 1.072572 |
| O | 0.207345 | -0.968672 | -1.577290 | H | -0.221640 | -3.513470 | 0.854088 |
| C | 2.065730 | -1.219479 | 0.605762 | H | 3.372413 | -2.917339 | 0.434561 |
| O | 0.973567 | -1.904703 | 1.053740 | H | 5.605362 | -3.364291 | 0.757830 |
| C | 0.799776 | -3.235581 | 0.585701 | H | 5.131667 | -3.497713 | -0.962918 |
| C | 3.285543 | -1.844579 | 0.334582 | H | 6.831568 | -3.166439 | -0.525023 |
| C | 4.386463 | -1.065491 | -0.035708 | H | 5.119688 | 0.921343 | -0.422611 |
| O | 5.619036 | -1.574697 | -0.315919 | - | - | - | - |

Table S6 Cytotoxic results of compounds **1−8** (concentration, 20 μM) against PATU8988T cell line

| compounds | reduction ratio |
| --- | --- |
| (+)-**1** | 43.5% |
| (−)-**1** | 40.2% |
| **2** | 91.4% |
| **3** | 36.7% |
| **4** | 25.0% |
| **5** | 30.6% |
| **6** | 33.3% |
| **7** | 65.5% |
| **8** | 72.1% |
